# Female cleaner fish cooperate more with unfamiliar males

**DOI:** 10.1098/rspb.2012.0063

**Published:** 2012-02-22

**Authors:** N. J. Raihani, A. S. Grutter, R. Bshary

**Affiliations:** 1 Genetics, Evolution and Environment, University College London, Gower Street, London WC1E 6BT, UK; 2 Institute of Zoology, Zoological Society London, Regent's Park, London, NW1 4RY, UK; 3 School of Biological Sciences, The University of Queensland, Queensland 4072, Australia; 4 Department of Zoology, Université de Neuchâtel, Rue Emilie Argand 11, 2007 Neuchâtel, Switzerland

**Keywords:** cleaning behaviour, cooperation, mutualism, Prisoner's Dilemma, punishment

## Abstract

Joint group membership is of major importance for cooperation in humans, and close ties or familiarity with a partner are also thought to promote cooperation in other animals. Here, we present the opposite pattern: female cleaner fish, *Labroides dimidiatus,* behave more cooperatively (by feeding more against their preference) when paired with an unfamiliar male rather than with their social partner. We propose that cooperation based on asymmetric punishment causes this reversed pattern. Males are larger than and dominant to female partners and are more aggressive to unfamiliar than to familiar female partners. In response, females behave more cooperatively with unfamiliar male partners. Our data suggest that in asymmetric interactions, weaker players might behave more cooperatively with out-group members than with in-group members to avoid harsher punishment.

## 1. Introduction

The prediction that players will cooperate more with familiar partners than with strangers has been supported in several empirical studies, both in humans and non-human species. For example, people are more likely to cooperate and more readily forgive defections when interacting with friends rather than strangers [1–4]. Similarly, close ties or familiarity with a partner are also thought to promote cooperation in other animals [5–7]. For example, among non-human primates, the mutual exchange of services and resources is often more common among closely bonded individuals than among more distantly connected individuals [5,8], while data from a laboratory study of zebra finches (*Taeniopygia guttata*) showed that individuals behaved more cooperatively with a familiar social partner than with an unknown partner [7].

Familiarity with a partner may increase cooperation if it is used as a proxy to judge the probability of future interactions and the associated likelihood of reaping a return on cooperative investments. Individuals with joint territorial boundaries, overlapping home ranges, or that belong to the same group will interact again in the future with a higher probability than individuals without stable territories or home ranges, or that meet during migration or belong to different groups. In the face of variation in the probability of re-encountering an interaction partner, individuals are expected to adapt their behaviour to the probability of future interactions (‘shadow of the future’; [9,10]). This is because cooperative interactions often involve investments, or behaviours that reduce the current payoff of the actor while increasing the payoff of a recipient [11,12]. The actor's initial investment may often be repaid by a reciprocal return investment from the recipient (or a bystander) [13,14] or, alternatively, because the actor's initial investment enables the recipient (or a bystander) to perform a self-serving response that benefits the actor as a by-product [15,16]. Return benefits are more likely to arise where actor and recipient expect to interact again in the future [13]. Therefore, familiarity with a partner is expected to promote cooperation because players may have an increased expectation that they will meet familiar partners again and therefore play as if in a repeated, rather than one-shot, game [1,17].

Partner familiarity might also support cooperation if familiar partners are more likely to have interdependent fitness [18], as may be the case with breeding partners or members of cooperative social groups, for example. Where fitness interests coincide in this way, individuals might behave more cooperatively with familiar partners because harming the partner to some extent also harms oneself. For example, in the aforementioned study using zebra finches, we note that though Prisoner's Dilemma payoffs were used (where defection yields higher immediate payoffs than cooperating, regardless of the partner's behaviour), social zebra finch pairs may have interdependent fitness [12]. This is because males and females often work together during a breeding season to raise young [19]. As singleton parents are highly unsuccessful (S. Griffith, unpublished data), cheating the partner may cause negative feedback to oneself [12]. If that is the case, then the payoffs in that laboratory experiment would approximate those of a Prisoner's Dilemma only with an unfamiliar partner. With a familiar partner, the payoffs would approximate those of the Prisoner's Delight game [20], where each player does best to cooperate, regardless of the partner's behaviour [18].

In the current study, we tested experimentally whether familiarity with a partner affected cooperation among mixed-sex pairs of bluestreak cleaner wrasse (*Labroides dimidiatus*)*.* Cleaners sometimes work in mixed-sex pairs to clean a joint client [21]. Although cleaner fish provide a service to reef fish clients by removing skin ectoparasites, they prefer to feed on client mucus. This preference results in a conflict of interest between cleaners and clients [22], and also between cleaners during joint client inspections [21,23]. Feeding on preferred mucus often leads to the termination of an interaction [24]. Therefore, as feeding against preference prolongs the interaction and hence the partner's access to the client, the extent to which cleaners feed against their preference is a measure of cooperative behaviour towards the co-inspecting partner [23]. In contrast, feeding on preferred food constitutes cheating the partner as it stops the latter's access to the client.

Cooperation within mixed-sex pairs of cleaner fish is based on asymmetric punishment [23,25] rather than positive reciprocity or other mechanisms (see [26]). Punishment occurs when a cheated individual pays a short-term cost to inflict harm on the cheating partner. Despite the initial cost, punishers may nevertheless benefit from their actions if the partner behaves more cooperatively in subsequent interactions [27,28]. Male cleaner fish are larger than and dominant to their female partners, and previous work using model Plexiglas ‘clients’ has shown that males aggressively punish females if females cheat during joint inspections [21,23,25]. Females provide a better service quality in response to male punishment, by feeding more against their preference [23,25]. This yields benefits to males because they can interact with joint clients for longer and thereby increase their food intake [23]. This feature of cooperative interactions among mixed-sex pairs of cleaner fish meant we could investigate how partner familiarity affected cooperation in a system where cooperation is based on asymmetric punishment, rather than positive reciprocity.

We consider two alternative hypotheses. Since punishment is an investment, individuals might be more likely to punish familiar partners if they expect to interact with these individuals more often in the future. Thus, in response to cheating, male cleaner fish might be more likely to punish familiar females. This would be particularly likely if the punishment yields long-term improved cooperative behaviour in the partner. Alternatively, if familiar partners have interdependent fitness then punishment might more often be inflicted on unfamiliar partners than on familiar individuals. This is because punishing an interdependent partner may to some extent also reduce the punisher's fitness. If established male–female cleaner fish pairs have interdependent fitness then it is possible that males will punish unfamiliar females more for cheating, especially if the benefits of punishment are accrued with short delay.

## 2. Methods

Data were collected at Lizard Island Research Station, Australia (14_40S, 145_28E) in 2010 and 2011. Each year, 12 established pairs of cleaner fish were caught with a barrier net and housed in pairs in aquaria (45 × 30 × 25 cm) for two to four weeks before experiments began. All cleaners were provided with a polyvinylchloride shelter tube (1 × 10 cm). Fish were trained to feed off Plexiglas plates (15 × 10 cm), which presented cleaners with the same foraging rules that they face when interacting with real clients. These Plexiglas ‘clients’ contained items of mashed prawn, or fish flakes mixed with prawn (hereafter ‘flake’), placed on them. Cleaners preferred prawn to flake but were trained that eating prawn led to plate removal. Thus, as with real clients, cleaner fish had to feed against their preference to continue interactions with model Plexiglas clients. All fish ate flake items before eating prawn items prior to commencing experiments (see [21] for more details and a schematic of the Plexiglas plate).

Twenty-four female cleaner fish were sequentially paired with (i) their familiar male partner and (ii) an unfamiliar male partner (treatment order was counterbalanced). Unfamiliar males were size-matched to the female's familiar male partner because previous work on this system has shown that males are more aggressive to similar-sized females [25]. This is because the bluestreak cleaner wrasse is a protogynous hermaphrodite, where female sex change appears to be prevented by the presence of a larger and more aggressive male [29]. Females that approach or exceed the current male in size may change sex, thereby becoming a reproductive competitor rather than remaining as a breeding partner. Male cleaners used in this study were on average 8.2 ± 0.1 cm (total length, mean ± se), while females were 7.0 ± 0.1 cm. The mean difference in size (size asymmetry) between familiar male–female partners was 1.1 ± 0.1 cm and the mean difference in size between familiar and unfamiliar male partners was 0.02 ± 0.1 cm. Data from one unfamiliar pair were not used in the analysis since the female was quite close in size to the new male partner (asymmetry = 0.5 cm) and immediately became dominant to the unfamiliar male. Thus, analyses are based on data from 45 pairs (24 familiar pairs and 23 unfamiliar pairs).

### (a) 2010 data collection

In 2010, each pair was observed for a total of 48 trials. There were two experimental treatments: (i) familiar partners (24 trials per couple) and (ii) unfamiliar partners (24 trials per couple). Experiments took place over four days, with 12 trials and a single treatment per couple per day. A trial consisted of a single plate presentation to a cleaner fish couple. Trials were separated by an interval of at least 30 min (maximum 60 min) to prevent satiation.

### (b) 2011 data collection

In 2011, we recorded foraging decisions of male and female cleaner fish with unfamiliar and familiar partners as we did in 2010, and also introduced a ‘separation’ treatment to assess the causal effect of male punishment on female foraging behaviour. In the separation treatment, males were prevented from punishing female partners by way of a temporary transparent partition. In the separation treatment, both fish could see one another and both still foraged on the same plate (as in [20]). In 2011, there were four experimental conditions: (i) familiar partners, together (16 trials per couple); (ii) familiar partners, separated (16 trials per couple); (iii) unfamiliar partners, together (16 trials per couple); and (iv) unfamiliar partners, separated (16 trials per couple). Experiments were conducted over eight days, with eight trials and a single treatment per couple per day. In 2011, each trial consisted of two plate presentations, which were separated by a 1 min interval. This design allowed us to investigate the short-term effects of male punishment on female foraging behaviour (as in [23]). As in 2010, trials were separated by a minimum interval of 30 min (maximum 60 min) to prevent satiation.

In both years, experiments took place between 08.00 and 17.00 h. Fish were not fed between trials, although in 2011, all fish were fed at the end of each experimental day. Since some data suggest that cleaners may behave more cooperatively (feed more against preference) when satiated (R. Bshary & A. Pinto, unpublished data), we expected that this additional feeding might mean that cleaners would be generally more cooperative in 2011 than in 2010. As such, we controlled for year in our statistical models, where appropriate. Treatment order was counterbalanced. In each presentation, a model Plexiglas client was made available to both members of the cleaner fish pair so that both individuals could begin foraging simultaneously. The model client was removed by the experimenter immediately after one cleaner ate a prawn item, signifying the end of the presentation. In each presentation, we recorded the number of flake items eaten by males and females, respectively, and the identity of the fish that ate the prawn item. In the 60 s following plate removal, we recorded the number of times the male chased the female as a measure of male aggression. This value was divided by the number of trials to obtain an aggression rate for each couple. Male aggression was not recorded during the separation treatment because males could not chase females.

#### (i) What factors affected female foraging behaviour?

First, we asked whether familiarity with the male partner affected female foraging behaviour (model 1). For each female, we calculated the ratio of flake items eaten per prawn item by summing the total number of flake items she ate and dividing by the total number of prawn items she ate over all presentations. This value was log-transformed and set as the response term in a linear mixed model (LMM) with normal error structure. Partner familiarity (familiar/unfamiliar), size asymmetry (cm), year (2010/2011) and treatment (separate/together) were included as potential explanatory terms. Model 1 was based on 70 data points from a total of 23 females (11 from 2010 and 12 from 2011).

Using a subset of the data (data collected in 2011), we then asked whether the intensity of male aggression following female cheating (prawn eating) in the first presentation affected female foraging behaviour in the second presentation (model 2). Since presentations one and two were separated by a 1 min interval, this model allowed us to assess the immediate effects of male aggression on female behaviour. Using instances where the female ate the prawn in the first presentation (*n* = 269 instances from 12 females), we created a binary response term where 1 = female ate prawn again in the second presentation and 0 = female did not eat prawn in the second presentation. This binary response term was set as the response term in a generalized LMM with binomial error structure. Male aggression following the first presentation, the size asymmetry between the pair and partner familiarity (familiar/unfamiliar) were included as explanatory terms.

Finally, we asked whether any effects of male aggression on female cheating persisted for more than 30 min by asking whether females were less likely to cheat in trial *n* + 1 following severe male aggression in trial *n* (model 3). Since trials were separated by a minimum of 30 min and a maximum of 60 min, this model allowed us to assess whether there were any longer-term effects of male aggression on female foraging behaviour. As in model 2, we used instances where females cheated in trial *n* but we had a larger sample size since data from both 2010 and 2011 could be used. Data from 2011 were restricted to trials where females cheated in the first presentation of trial *n.* Based on 509 cheating events (23 females), we created a binary response term where 1 = female cheated again in trial *n* + 1 and 0 = female did not cheat again in trial *n* + 1. This binary response term was set as the response term in a generalized LMM with binomial error structure. Male aggression, the size asymmetry between the pair and partner familiarity (familiar/unfamiliar) and year (2010/2011) were included as explanatory terms. In models 1–3, male and female identities were included as random terms to control for the effects of repeated measures on the distribution of the data.

#### (ii) What factors affected male punishment of cheating females?

We asked whether male aggression was associated with partner familiarity (model 4). Male aggression (chases per second) was log-transformed and set as the response term in an LMM with normal error structure. Partner familiarity (familiar/unfamiliar), year (2010/2011) and size asymmetry were included as explanatory terms, and male and female identities were included as random terms. Model analysis was based on 46 data points from 23 females (11 from 2010 and 12 from 2011). The sample size for this model is smaller than the sample size for model 1 because data from the separation treatment were not included.

#### (iii) What factors affected male foraging behaviour?

We then investigated how familiarity with the female partner affected male foraging behaviour (model 5). The ratio of flake to prawn items eaten by males was calculated in the same way as for females (total number of flake items eaten/total number of prawn items eaten). This value was log-transformed and set as the response term in an LMM with normal error structure. We included partner familiarity (familiar/unfamiliar), size asymmetry and year (2010/2011) as fixed terms. Male and female identities were included as random terms in the model to control for the effects of repeated measures on the distribution of the data. Data from one male were also excluded from the final model, since it ate an extremely high ratio of flake to prawn items (flake to prawn ratio 49 compared with a mean of 4.2 ± 0.4), which significantly affected the fit of the model to the data and resulted in a violation of the assumption of normally distributed residuals. The model analysis was, therefore, based on 45 data points from 26 males.

Summarized details of all models are presented in table 1. Data were analysed with R v. 2.8.1 (www.r-project.org). Tests were two-tailed and data were checked and transformed where necessary to ensure they satisfied the assumptions of statistical tests. Models were run by initially including all terms in a ‘full’ model and then sequentially dropping explanatory terms, retaining only those whose removal resulted in a significant loss of predictive power. The significance of dropped terms was obtained by adding them to the resultant ‘minimal’ model. All two-way interactions were checked but only presented where *p* < 0.05.

**Table 1.** Description of analyses.

model	question	response term	explanatory terms	data used
1	Did familiarity with male partner affect female foraging behaviour?	log (ratio of flake items per prawn item eaten by female)	partner familiarity (familiar/unfamiliar)	*n* = 70 data points from 2010 and 2011
			size asymmetry (cm)	
			treatment (separate/together)	
			year (2010/2011)	
2	Did intensity of male aggression affect probability that female would cheat again (short-term)?	female cheating (1 = cheated again in P2; 0 = did not cheat again in P2)	male aggression following P1 (chases/trial)	*n* = 269 instances of female cheating from 2011 only
			partner familiarity (familiar/unfamiliar)	
3	Did intensity of male aggression affect probability that female would cheat again (long-term)?	female cheating (1 = cheated again in trial *n* + 1; 0 = did not cheat again in trial *n* + 1)	male aggression (chases/trial)	*n* = 509 instances of female cheating from 2010 and 2011
			partner familiarity (familiar/unfamiliar)	
			size asymmetry (cm)	
4	Did male aggression vary with familiarity of female partner?	log (male aggression)	partner familiarity (familiar/unfamiliar)	*n* = 46 data points from 2010 and 2011
			size asymmetry (cm)	
			year (2010/2011)	
5	Did familiarity with female partner affect male foraging behaviour?	log (ratio of flake items per prawn item eaten by male)	partner familiarity (familiar/unfamiliar)	*n* = 45 data points from 2010 and 2011
			size asymmetry (cm)	
			year (2010/2011)	

## 3. Results

### 

#### (i) What factors affected female foraging behaviour?

**Figure 1.**
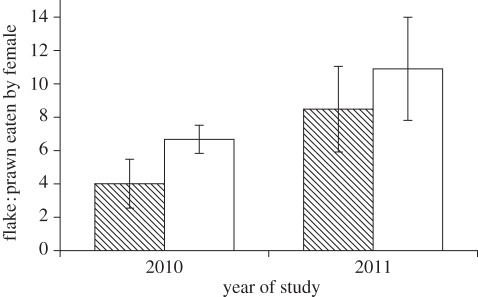
Ratio of flake to prawn items (±s.e.) eaten by female cleaner fish when paired with a familiar and unfamiliar male partner, according to the year when the data were collected. Mean values were generated from raw data and so do not control for other terms in the model. Shaded bars, familiar partners; open bars, unfamiliar partners.

**Figure 2.**
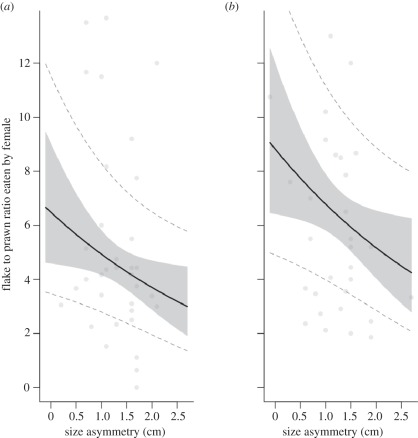
Ratio of flake to prawn items eaten by female with (*a*) familiar male partners and (*b*) unfamiliar male partners according to size asymmetry (centimetres) within the pair. The thick black lines were generated from predictions based on the minimal model. The grey shaded area represents the standard error associated with the fixed effect (partner familiarity). Dashed lines are the standard error associated with the predictions for the random terms in the model. Points were generated from raw data.

**Figure 3.**
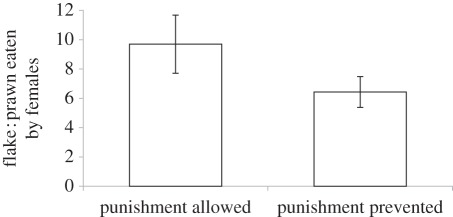
Ratio of flake to prawn items eaten (±s.e.) by female cleaner fish when male punishment was allowed and when it was prevented. Means were generated from raw data.

Females ate a higher ratio of flake to prawn items when paired with an unfamiliar male partner (model 1: *χ*^2^ = 5.2, *p* = 0.02; figures 1 and 2) and ate a lower ratio of flake to prawn items when male punishment was prevented (model 1: *χ*^2^ = 4.1, *p* = 0.04; figure 3). The significant negative effect of size asymmetry on the ratio of flake to prawn items eaten by females (model 1: *χ*^2^ = 5.4, *p* = 0.02, figure 2) was controlled for in the above results. We also found that females were generally more cooperative (ate more against their preference) in the second year of this study (model 1: *χ*^2^ = 4.2, *p* = 0.04; figure 1). The tendency for females that cheated in the first presentation to cheat again in the second presentation was negatively associated with the intensity of male punishment (model 2: *χ*^2^ = 3.92, *p* = 0.048). There were no significant effects of size asymmetry (model 2: *χ*^2^ = 0.57, *p* = 0.45) or partner familiarity (model 2: *χ*^2^ = 0.06, *p* = 0.80) on female propensity to cheat again. There were no significant lasting effects of male aggression on female propensity to cheat again in the next trial (model 3: *χ*^2^ = 0.40, *p* = 0.53), although females were generally more likely to cheat again in the first year of this study than in the second year (model 3: *χ*^2^ = 6.72, *p* = 0.01).

#### (ii) What factors affected male aggression against female partners?

**Figure 4.**
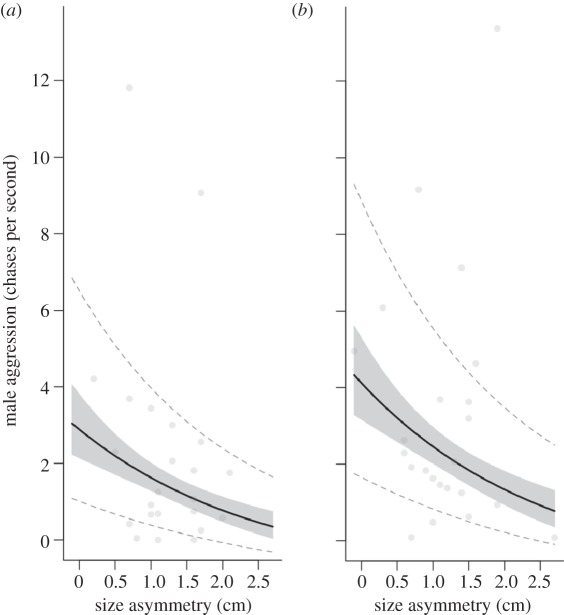
Male aggression (chases per second) against (*a*) familiar female partners and (*b*) unfamiliar female partners according to the size asymmetry (centimetres) within the pair. The thick black lines were generated from predictions based on the minimal model. The grey shaded areas represent the standard error associated with the fixed effect (partner familiarity). Dashed lines are the standard error associated with the predictions for the random terms in the model. Points were generated from raw data.

Females received more aggression from unfamiliar males than from familiar male partners (model 4: χ^2^ = 9.32, *p* = 0.002; figure 4), after controlling for the negative effect of size asymmetry on male aggression (model 4: *χ*^2^ = 7.08, *p* = 0.008, figure 4). There was no significant effect of year during which data were collected on male aggression (model 4: *χ*^2^ = 2.76, *p* = 1.0).

#### (iii) What factors affected male foraging behaviour?

Unlike females, familiarity with a partner did not seem to affect male foraging decisions. The ratio of flake to prawn items eaten by males was not significantly affected by partner familiarity (model 5: *χ*^2^ = 0.11, *p* = 0.74), by the size asymmetry within the pair (model 5: *χ*^2^ = 1.34, *p* = 0.25), or by the year during which the data were collected (model 5: *χ*^2^ = 3.46, *p* = 0.062).

## 4. Discussion

During joint inspections with model clients, female cleaner fish cooperated more (by feeding more against their preference) with unfamiliar than with familiar male partners. Female cooperative behaviour can be explained by asymmetric punishment in response to cheating in this species. Males were more aggressive to unfamiliar female partners and female propensity to cheat again decreased with increasing severity of male punishment. Furthermore, females behaved less cooperatively (with both familiar and unfamiliar males) when male aggression was prevented. Asymmetric punishment may also explain why male behaviour was unaffected by the familiarity of the female partner. Female cleaner fish are subordinate to males and do not punish male partners for cheating [23]. Since punishment appears to be a key mechanism that causes cleaner fish to behave more cooperatively [23,25,30], male foraging behaviour may be unaffected by partner familiarity *per se*.

The fact that males were more aggressive to unfamiliar females than familiar females may initially seem counterintuitive. Punishment is an investment, the benefits of which can be gained through future interactions with the punished individual [27,28,31]. Since familiar partners may be more likely to interact in the future than unfamiliar partners, we could predict that individuals would be more likely to punish familiar partners. Instead, we found the opposite pattern. This pattern may arise because, in this system, the benefits to males of punishing females (in terms of increased female cooperative behaviour) arise immediately. Interactions with clients are extremely frequent (approx. 2300 interactions per day, [32]), meaning that males may benefit from increased female cooperative behaviour very soon after inflicting the punishment. Moreover, the effects of punishment are short-lived: we found no significant effect of male punishment intensity on female propensity to cheat again 30–60 min after the punishment. Thus, in this system, males may gain no additional benefit, in terms of increased probability of future interactions, from punishing familiar rather than unfamiliar females.

Instead, interdependencies in fitness could mean that males experience lower net benefits when they punish familiar females, thus explaining why males were more aggressive to unfamiliar females in this study. We suggest that the costs to male cleaner fish of female cheating might vary according to whether the female is a social partner or not. Specifically, males might experience lower costs when familiar, rather than unfamiliar, female partners cheat if the extra energy gained by cheating females translates into the production of extra eggs. If cheating by familiar females is less costly to their male partners, selection may favour decreased investment in punishment. Similar interdependencies in fitness have also been invoked to explain the patterns of cooperation observed in captive zebra finches [7,12]. Variation in the costs experienced by males when females cheat might also explain why male punishment of cheating females varied with the size asymmetry within the pair. In this species, larger females pose a reproductive threat to males since they may be more likely to change sex [29]. Accordingly, here and previously [25], we have shown that males are more likely to punish relatively large females that cheat, while remaining more tolerant of cheating by smaller females. In this study, we found that relatively small females were also less cooperative—this may be a direct consequence of the fact that small females that cheated tended to receive less severe aggression from males.

An alternative (and non exclusive) explanation for the observed pattern of increased male aggression towards unfamiliar female partners is that males use aggression to establish dominance over unfamiliar females. As such, males may initially be very aggressive towards unfamiliar females and this might precipitate increased cooperative behaviour from female cleaners. If this were the case then we would expect male aggression in response to female cheating to diminish over time, though we did not collect data for long enough to test this idea.

In general, we predict that in interactions where players do not have interdependent fitness, individuals will be more likely to invest either to benefit or to harm partners when partners are familiar. This is because, all else being equal, familiar partners may be more likely to interact frequently and so individuals stand a greater chance of reaping a return on their initial investment. This prediction holds even in cases where the benefits of investment arise through the self-serving behaviour of the partner (pseudo-reciprocity) rather than through costly return investments (reciprocity). This is because individuals might be most likely to reap the benefits associated with investing in helping or harming a partner if they expect to meet the partner again in the future or when association with the partner allows them to benefit from the partner's self-serving behaviour. Conversely, the predictions differ in interactions where players’ fitness interests coincide to some extent. Here, individuals might be more likely to invest in familiar partners when the investment benefits the partner. However, where the investment harms the partner (as is the case with punishment) then individuals might instead invest more in punishing unfamiliar partners.

In humans, so-called parochialism refers to the tendency for people to punish out-group members more severely than in-group members for cheating [33–36]. Hostility towards out-group members is expected to go hand in hand with increased cooperative behaviour towards in-group individuals and may have been selectively advantageous in inter-group conflicts [33,35,37], but see [38]. The data we present here in cleaner fish resemble such parochialism in a rudimentary form. However, in our experiment, male cleaner fish were more hostile towards unfamiliar females without being more cooperative towards familiar female partners. Moreover, as a consequence of more severe aggression, females were more cooperative with unfamiliar males than with their established male partner. Our results are not directly comparable to the human studies because the latter are typically designed in such a way that punishment can be administered only by bystanders and games usually consist of only one round [33]. It would be interesting to test how humans and other species behave in a game that fits our experiment. We suggest that in asymmetric repeated interactions where cooperation is based on self-serving punishment, weaker players might behave more cooperatively with outsiders than with in-group members to avoid harsher punishment.
